# Schneiderian Membrane Collateral Damage Caused by Collagenated and Non-Collagenated Xenografts: A Histological Study in Rabbits

**DOI:** 10.3390/dj11020031

**Published:** 2023-01-26

**Authors:** Yasushi Nakajima, Daniele Botticelli, Ermenegildo Federico De Rossi, Vitor Ferreira Balan, Eduardo Pires Godoy, Erick Ricardo Silva, Samuel Porfirio Xavier

**Affiliations:** 1 Department of Oral Implantology, Osaka Dental University, 8-1 Kuzuhahanazonocho, Hirakata 573-1121, Osaka, Japan; 2 ARDEC Academy, 47923 Rimini, Italy; 3 Department of Oral and Maxillofacial Surgery and Periodontology, Faculty of Dentistry of Ribeirão Preto, University of São Paulo, Ribeirão Preto 14040-904, Brazil; 4 Department of Oral Biology, Faculty of Dentistry of Ribeirão Preto, University of São Paulo, São Paulo 05508-060, Brazil

**Keywords:** animal study, sinus floor elevation, bone healing, Schneiderian membrane, histology

## Abstract

Background: The Schneiderian membrane (SM) that is in contact with biomaterial granules may become thinner and eventually perforate. It has been shown that these events are related to the biomaterial used. Hence, the main aim of the present study was to compare the damaging effects of two xenografts with different resorbability rates on SM. The secondary aim was to evaluate the possible protection from damage offered by a collagen membrane placed adjacent to the SM and by inward displacement of the bone window with the SM during elevation. Methods: Thirty-six albino New Zealand rabbits underwent bilateral sinus elevation. One group of 18 animals received deproteinized bovine bone mineral (DBBM group) and the other received swine-collagenated corticocancellous bone (collagenated group). Moreover, in the DBBM group, the bone window was displaced inward during elevation in one sinus together with the SM. In the collagenated group, a collagen membrane was placed adjacent to the SM in one sinus. Six animals were assessed per period after 2, 4, and 8 weeks. Results: The mean pristine mucosa width ranged between 67 µm and 113 µm, and none had a width of <40 µm. In the 2-week group, the elevated mucosa of the DBBM group presented 59 thinned sites and five perforations, while in the collagenated group, 14 thinned sites and one perforation were observed. Damage to SM decreased in number in the 4-week treatment group. In the 8-week group, the number of thinned sites in the DBBM group increased to 124, and the perforations to 8. In the collagenated group, 7 thinned sites and 1 small perforation were observed. Conclusions: More damage to the Schneiderian membrane was observed in the DBBM group than in the collagenated group. The presence of the inward bone window offered protection from damage to the Schneiderian membrane.

## 1. Introduction

When sufficient bone volume for implant installation is not available in the posterior segments of the maxilla, sinus floor elevation has been proven to be a reliable method for bone augmentation. Both lateral [1,2] and transcrestal approaches [3] are widely used, showing a high rate of success [4,5,6]. Intra-surgical complications may occur, among which perforation of the Schneiderian membrane (SM) is the most frequent [7,8,9,10]. The thickness of the mucosa (<0.8 mm) and presence of the septa are considered risk factors for perforations [9,10]. Extrusion of biomaterials into the sinuses has been reported both during the surgical procedure [11,12] and at a later stage, sometimes requiring graft removal [13,14,15]. Depending on its dimension, the perforation might be left healed as it is or treated with different techniques, the most used being the placement of a collagen membrane [16]. Under such conditions, perforation of the SM was not considered a risk factor for implant survival in a systematic review [16]. While these perforations are referred to as a consequence of surgery, damage to the SM has been reported in the subsequent healing periods. This damage, represented by thinning of the SM and perforations, has been observed in a rabbit model. It has been shown that damage occurs in the mucosa in contact with granules of biomaterial [17,18,19,20] or even with implant apexes and threads [18,21]. It has also been shown that grafts with a lower resorption rate present more damage than those with a higher resorption rate [17,18,19,20]. The results of these experiments suggest that resorption and surface characteristics might play an important role in the possible damage to the SM. This justifies further evaluation to identify the biomaterial that causes the least damage to the SM. Hence, the main aim of the present study was to compare the damaging effects of two xenografts with different resorbability rates on SM. The secondary aim was to evaluate the possible protection from damage offered by a collagen membrane placed adjacent to the SM and by inward displacement of the bone window with the SM during elevation.

## 2. Materials and Methods

### 2.1. Ethical Statements

Two experiments were selected to evaluate the damage to the Schneiderian membrane (SM) triggered by contact with the residual granules of collagenated or non-collagenated xenografts. The experimental protocols were submitted to and approved by the Ethical Committee of the Faculty of Dentistry of Ribeirão Preto, University of São Paulo (collagenated study: 2015.1.834.58.7, approved on 18 November 2015 [22]; not-collagenated study: protocol No. 2017.1.278.58.9, approved on 14 June 2017 [23]). The article was written in accordance with the ARRIVE guidelines. Brazilian rules for animal care were accurately followed.

### 2.2. Study Design

The maxillary sinuses were augmented bilaterally in both studies in rabbits using either a collagenated xenograft (collagenated group) [22] or a non-collagenated deproteinized bovine bone mineral (DBBM group) [23]. Sinus mucosa thinning and perforations were assessed, and the results of the two groups were compared. Histomorphometric data describing healing within elevated regions have been reported elsewhere [22,23].

### 2.3. Experimental Animals

Thirty-six albino New Zealand rabbits, 18 in each experiment, ~3.4–4 kg of weight and 4–6 months old, were used. Three groups of six animals were formed in each experiment and euthanized 2, 4, or 8 weeks after surgery.

### 2.4. Biomaterials

Collagenated group: The xenograft was composed of collagenated corticocancellous bone obtained from swine at temperatures up to 130 °C (Gen-Os, 0.250–1.0 mm, OsteoBiol, Tecnoss, Giaveno, Italy). The total and intraparticle porosities are 33.1% and 21%, respectively. The real density was 2.43 g/cm^3^ and the mineral content was 64.6% [24]. The membrane was obtained from equine collagen (Evolution, 0.3 mm, OsteoBiol, Tecnoss).

DBBM group (non-collagenated): the xenograft was composed of a deproteinized cancellous bone obtained at a temperature of up to 300 °C from bovine (Bio-Oss^®^ granules 0.250–1.0 mm; Geistlich Biomaterial, Wolhusen, Switzerland). The total and intraparticle porosities are 63.5% and 51%, respectively. The real density was 3.21 g/cm^3^ and the mineral content was 95% [24]. The membrane was made of bilayer porcine collagen (Bio-Gide, Geistlich Biomaterial, Wolhusen, LU, Switzerland).

### 2.5. Sample Size

Sample size calculation was based on data reported in a previously published article [18]. The number of thinning mucosal sites in contact with the biomaterials after 40 days of healing was used. The effect size was estimated to be 2.983. By applying α = 0.05 and a power of 0.9, four animals in each group were calculated. Given that a lower rate of resorption is expected for the collagenated graft used in the present study than for the autogenous bone used in the study used for sample calculation [18], six instead of four animals in each group were judged to be sufficient to reject the null hypothesis that the population means of the two groups were equal.

### 2.6. Randomization and Allocation Concealment

The randomization was carried out electronically by an author not involved in the surgery. The allocation treatment was maintained in sealed opaque envelopes and revealed after the opening of the access windows in the non-collagenated group and after the exposure of the nasal bone in the DBBM group. The assessor of the histology (E.F.D.R.) was not informed before the histological evaluation regarding the intention of comparing the data between the two studies.

### 2.7. Clinical Procedures

Similar methods for anesthesia were adopted. Briefly, acepromazine (1.0 mg/kg, Acepran^®^, Vetnil, Louveira, São Paulo, Brazil) subcutaneously and xylazine (3.0 mg/kg, Dopaser^®^, Hertape Calier, Juatuba, Minas Gerais, Brazil) and ketamine hydrochloride (50 mg/kg, Ketamin Agener, União Química Farmacêutica Nacional S/A, Embu-Guaçú, São Paulo, Brazil) intramuscular were administrated. Local anesthesia was also administered at the experimental site.

The muzzle of each rabbit was shaved and disinfected. Skin and periosteum on the nasal dorsum and the bone were disclosed. Trephines and drills were used to prepare the bilateral access windows. As a reference for the histological process, a small screw was placed into the nasal-incisal suture between the two osteotomies.

**Figure 1:**
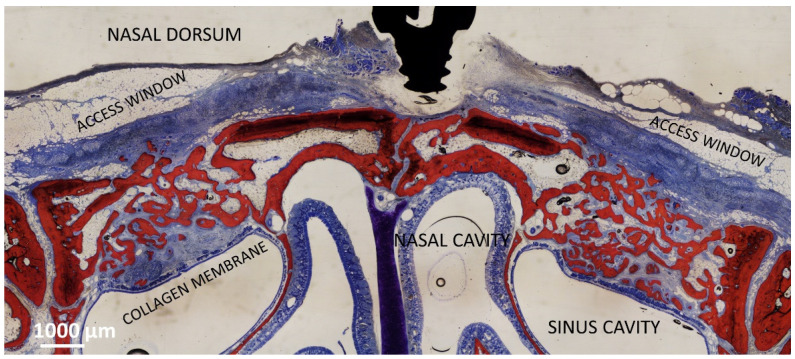
Photomicrograph showing the healing after 8 weeks of sinuses of the collagenated group. On the right is the control group, on the left the site with the collagen membrane adjacent the sinus mucosa. The collagen membrane is not completely resorbed yet.

In the collagenated study [22], a collagenated xenograft was used (Gen-Os, 0.250–1.0 mm, OsteoBiol, Tecnoss). In one sinus of each rabbit, a collagen membrane (Evolution, 0.3 mm, OsteoBiol, Tecnoss) was placed adjacent to the SM before the placement of the xenograft (collagenated Mb). No membrane was placed beneath the SM in the opposite sinus (collagenated No-Mb). Both osteotomies were covered with collagen membranes of the same type (see the histological outcome after 8 weeks in Figure 1).

**Figure 2:**
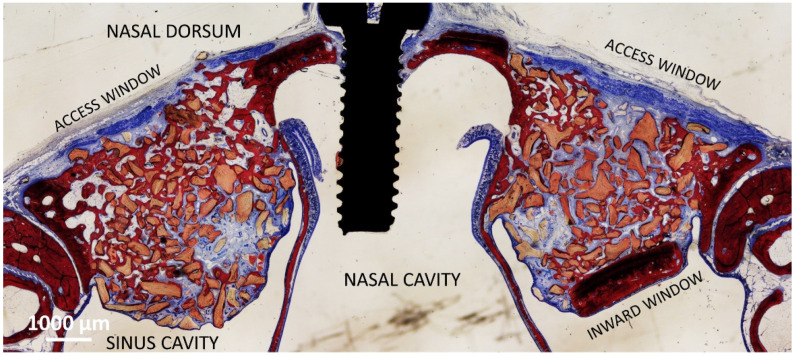
Photomicrograph showing the healing after 8 weeks of sinuses of the DBBM group. On the left is the control group, on the right the site with the bone window displaced inward.

In the DBBM group [23], a trap door technique was adopted at the test site so that a bone window was prepared and displaced inward (DBBM Wn), whereas at the control sites, the bone lid was removed (DBBM No-Wn). Both elevated spaces were filled with deproteinized bovine bone mineral (DBBM; Bio-Oss^®^ granules 0.250–1.0 mm; Geistlich Biomaterial). Both access windows were covered with a collagen membrane (Bio-Gide, Geistlich Biomaterial, Wolhusen), and the wounds were subsequently sutured (see the histological outcome after 8 weeks in Figure 2).

### 2.8. Euthanasia

The rabbits were anesthetized and subsequently euthanized with an overdose of sodium thiopental (1.0 g, 2 mL, Thiopentax^®^, Cristália Produtos Químicos Farmacêuticos, Itapira, São Paulo, Brazil).

### 2.9. Housing and Husbandry

The animals were housed in individual cages in a climatized room with ad libitum access to food and water. The biological functions and wounds were checked daily by specialized operators during the entire experimental period. Oxytetracycline dihydrate, ketoprofen, and tramadol or buprenorphine were administered [22,23].

### 2.10. Histological Preparation

The biopsies were maintained in 10% buffered formalin and, subsequently, dehydrated in increasing concentrations of ethanol and then included in resin (LR White^TM^ hard grid, London Resin Co. Ltd., Berkshire, UK). Thermal polymerization was performed in an oven at 60 °C for 24 h. Using a precision slicing machine (Exakt, Apparatebau, Norderstedt, Germany), the specimens were first cut in the coronal plane crossing the small screw used as a reference for histological processing. Subsequently, two slices of approximately 150 µm were prepared and reduced to approximately 60 µm using a grinding machine (Exakt, Apparatebau, Norderstedt, Germany). The histological slides were stained with either toluidine blue or Stevenel’s blue and Alizarin red.

### 2.11. Calibration for Histometric Evaluations

All measurements were performed by an author (E.F.D.R.). Before starting the measurements, calibration with another professional (D.B.) was performed until the inter-rater agreement achieved a Cohen’s coefficient of k > 0.80.

### 2.12. Experimental Outcomes and Statistical Methods

The pristine mucosa was measured at the medial and lateral sinus walls in regions not included in the elevated space. The mean values of the two measurements were used.

Elevated mucosa with a width of <40 µm was considered thinned. All sites with thinned mucosa were measured and registered. The number and dimensions of the SM perforations in the graft granules were also assessed.

Pristine and thinned mucosae were evaluated on slides stained with Stevenel’s blue and alizarin red, while perforations were also evaluated on slides stained with toluidine blue. As the two slides were taken from the central region of the elevated space, the same perforation might have been included in both histological slides. In this case, only one perforation was considered.

The primary variables were the number of thinned mucosa and perforations. Intra-group comparisons were performed, that is, DBBM Wn vs. DBBM No-Wn and collagenated Mb vs. collagenated No-Mb. The differences between DBBM No-Wn and collagenated No-Mb were also analyzed. Differences between weeks 2 and 4 and between weeks 4 and 8 were also tested.

The width of the pseudostratified epithelium was also a primary variable. The differences between thinned and pristine sites as well as differences between 2 and 4 weeks and 4 and 8 weeks of healing were analyzed.

The Shapiro–Wilk test was used to test the normal distribution of the data. The paired *t*-test or Wilcoxon test was used for dependent variables, while the unpaired *t*-test of the Mann–Whitney test was applied for independent variables. Software Prism 9.4.1 (GraphPad Software, LLC, San Diego, CA, USA) was used for statistical analyses.

## 3. Results

### 3.1. Clinical Outcomes

In the collagenated study, two Schneiderian membrane (SM) perforations of <1 mm were observed during elevation, one at a membrane site in the 8-week period, and one in a control site in the 4-week period. The control site was left untreated. Perforations were not observed in the DBBM group. No further complications were observed in any of the groups. All histological slides were available for analysis, with *n* = 6 for all groups and periods.

### 3.2. Descriptive Histological Evaluation

Thinned mucosa sites (<40 µm) were identified in all periods and were mostly present in the DBBM group. In this group, the thinned sites decreased between 2 and 4 weeks and increased during the 8-week period. In the collagenated group, the thinned sites were found in low numbers during all periods evaluated.

**Figure 3:**
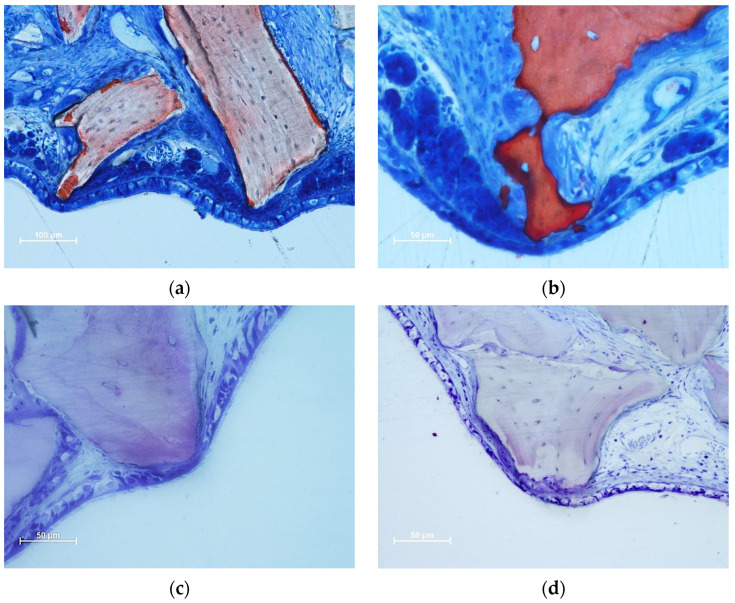
Photomicrographs of ground sections illustrating thinned mucosa sites. (**a**) DBBM site after two weeks of healing. Displacement and deformation of the mucosal glands and vessels were observed. Osteoclasts around the granules showed little efficacy in resorbing. (**b**) Collagenated site after 2 weeks of healing. Displacement and deformation of the mucosal glands and vessels were also visible in this image. The pseudostratified epithelium was reduced in width and presented a loss of globular cells and a reduction in the height of the cilias. Strong osteoclastic activity was observed around collagenated xenografts. (**c**,**d**) DBBM sites after 4 weeks of healing. Progressive thinning of the mucosa and pseudostratified epithelium was observed in the most advanced cases. (**a**,**b**) Stevenel’s blue and alizarin red staining; (**c**,**d**) toluidine blue staining.

The thinned mucosa sites were in contact with the remains of granules, especially on sharp edges protruding beyond the periphery of the dome-shaped elevated sinus. In the initial cases, only the lamina propria and its structures were involved. The thinned sites were characterized by the displacement and deformation of the vessels and mucosal glands (Figure 3a,b). In more advanced cases, the pseudostratified epithelium presented a decreased width and loss of globet cells and cilias (Figure 3c,d). In the latest stages, only a thin layer of cells or connective tissues separates the granules from the sinus cavity. The thinned sites were generally devoid of inflammatory infiltrates.

**Figure 4:**
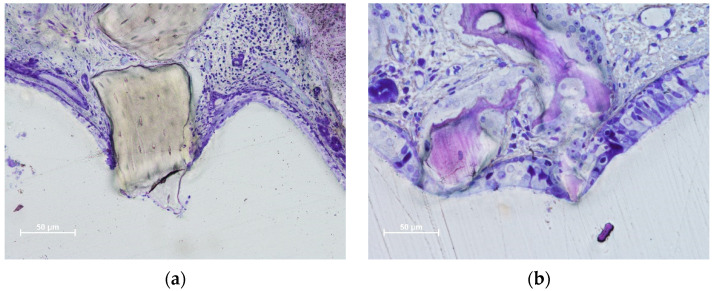

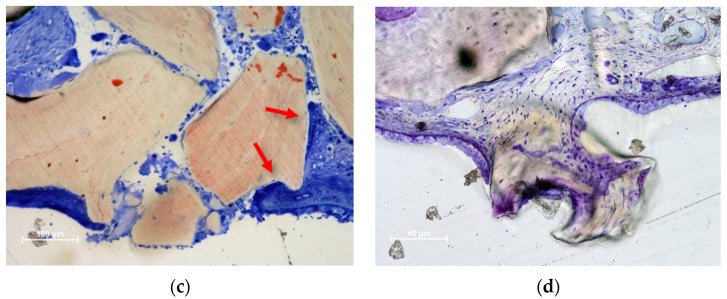
Photomicrographs of ground sections illustrating perforations of the Schneiderian membrane. The granules protruding towards the sinus cavity presented a tapered epithelium surrounding the perforation. (**a**) Eight-week healing. DBBM granule protruding through the SM and presenting an inflammatory infiltrate. (**b**) Collagenated granules. A very small perforation was found after 8 weeks of healing. The residual granules are under strong resorption. (**c**) DBBM granules trespassing the sinus mucosa in a 2-week period specimen. Note the epithelium surrounding one granule (red arrows). (**d**) DBBM granules almost ejected outside the elevated space. (**a**,**b**,**d**) Toluidine blue staining; (**c**) Stevenel’s blue and alizarin red staining.

In the DBBM group, some perforations were observed in the 2-week period, which decreased in number after 4 weeks. After eight weeks, the number of perforations increased at the DBBM No-Wn sites. The granules protruding towards the sinus cavity presented a tapered epithelium surrounding the perforation, showing a possible attempt to separate the internal environment from the sinus cavity (Figure 4a–c). Inflammatory infiltrates and tissue disruptions are often observed. In the collagenated group, one perforation was observed after 2 weeks in the collagenated Mb sites, no perforations were observed after 4 weeks, and one very small perforation was found in the collagenated No-Mb sites after 8 weeks of healing (Figure 4d).

### 3.3. Histometric Assessments

**Figure 5:**
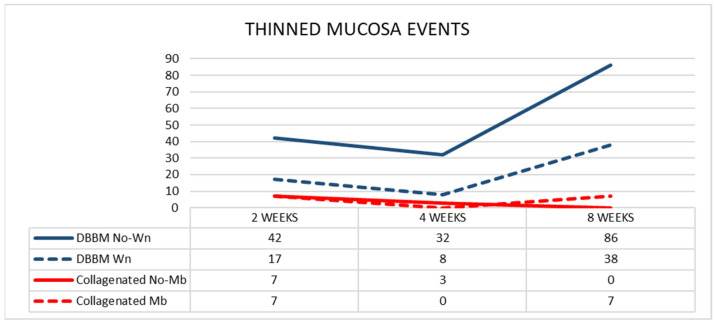
Graph illustrating the number of thinned mucosa sites presenting a width < 40 µm in the various groups.

The mean pristine mucosa width ranged between 67 µm and 113 µm, and none had a width of <40 µm. After 2 weeks of healing, in the DBBM group, 17 thinned mucosae (mean width 24 µm) in the Wn sites, and 42 thinned mucosae (mean width 25 µm) in the No-Wn sites were found (Figure 5). In the collagenated group, seven thinned mucosae were found at both Mb and No-Mb sites, with a mean width ranging from 19–26 µm. No statistically significant differences were found in any of the variables analyzed.

After 4 weeks of healing, in the DBBM group, eight and 32 thinned mucosae were found in the Wn and No-Wn sites, respectively (mean mucosa with 22 µm in both). In the collagenated group, three thinned mucosae were observed in the No-Mb sites (mean width 28 µm) and none in the Mb sites. Statistically significant differences were only found between DBBM Wn and No-Wn and between DBBM No-Wn and collagenated No-Mb. No statistically significant differences were found between 2 and 4 weeks of healing.

After 8 weeks of healing, in the DBBM group, the thinned mucosa sites increased in number to 38 (mean width 23 µm) in the Wn sites, and to 86 (mean width 19 µm) in the No-Wn sites. In the collagenated group, seven thinned mucosae were found in the Mb sites (mean width 15 µm) and none in the No-Mb sites. Statistically significant differences were only found between DBBM Wn and No-Wn and between DBBM No-Wn and collagenated No-Mb. Statistically significant differences between 4 and 8 weeks were found only in the DBBM group for both Wn and No-Wn sites.

**Figure 6:**
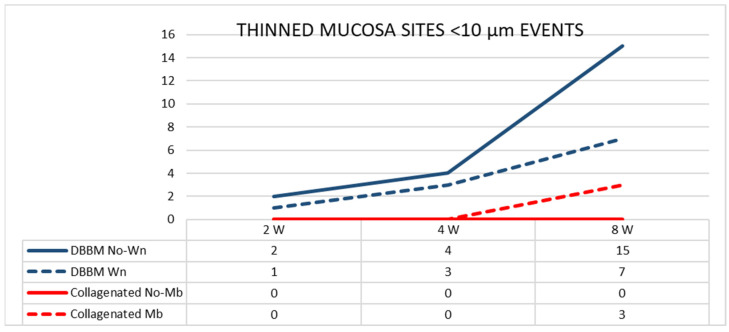
Graph illustrating the number of thinned mucosa sites presenting a width < 10 µm in the various groups evaluated in three different periods.

The number of sites with a width < 10 µm increased between 2 and 8 weeks in the DBBM group, whereas a slight increase in number was observed in the Mb sites of the collagenated group (Figure 6).

**Figure 7:**
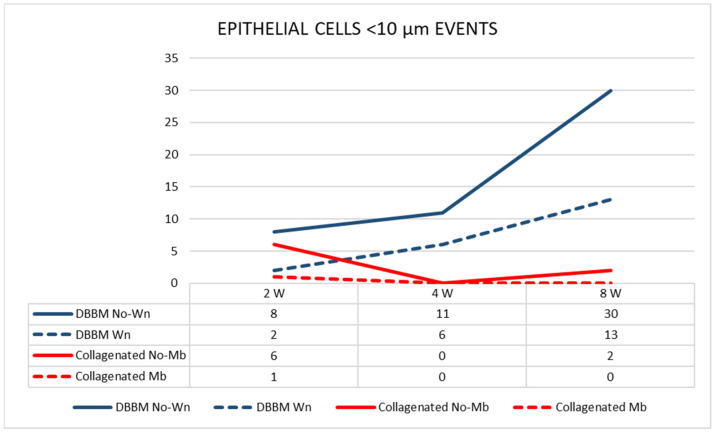
Graph illustrating the number of pseudostratified epithelium sites presenting a width < 10 µm in the various groups evaluated in three different periods.

The pseudostratified epithelium in the thinned mucosae in the DBBM group was thinner than that in the pristine mucosa. The differences were statistically significant at two and eight weeks after healing. The number of sites with an epithelium width of <10 µm increased over time in the DBBM group (Figure 7).

**Figure 8:**
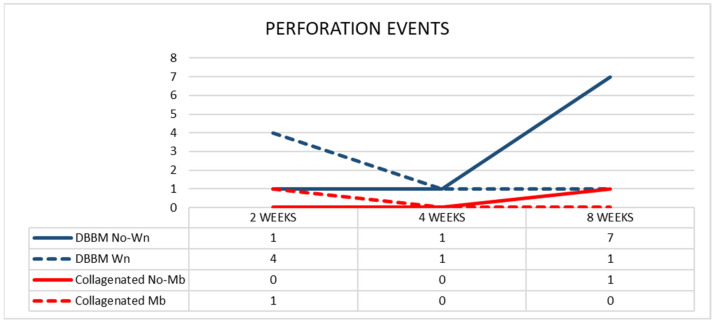
Graph illustrating the number of perforations in the various groups evaluated in three different periods.

After two weeks of healing, in the DBBM group, four perforations were distributed in four sinuses (mean dimension 375 µm) in the Wn sites, and one perforation was found in the No-Wn sites (143 µm in dimension) (Figure 8). In the collagenated group, one perforation was found at the Mb sites (35 µm in dimension) and none at the No-Mb sites.

After four weeks of healing, in the DBBM group, one perforation was found in the Wn sites (42 µm in dimension) and one in the No-Wn sites (35 µm in dimension). No perforations were observed at either site in the collagenated group. After 8 weeks of healing, one perforation in the Wn sites (134 µm in dimension) and seven in the No-Wn sites (mean dimension 89 µm) were observed in the DBBM group. In the collagenated group, one perforation at the No-Mb sites (7 µm in dimension) and no perforations at the Mb sites were observed. No statistically significant differences were found among the sites and healing times.

## 4. Discussion

The aim of the present study was to compare the damage caused by contact of the Schneiderian membrane (SM) with granules of collagenated and non-collagenated (deproteinized bovine bone mineral; DBBM) xenografts. It was observed that this contact produced thinning (<40 µm) of the SM compared to the width of the non-elevated pristine mucosa (range 67–113 µm). Moreover, these sites generated thinning of the pseudostratified epithelium and perforations of the SM in both groups. After eight weeks of healing, significantly thinner mucosa sites were found in the DBBM group than in the collagenated group. A higher number of perforations was observed in the DBBM No-Wn sites than in the other sites. However, this difference was not statistically significant.

Thinned mucosae were characterized by a reduction in width, presenting displacement and deformation of the mucosal glands and vessels (Figure 3a,b). The pseudostratified epithelium in the thinned mucosa was also reduced in width (Figure 3c,d). SM perforations were observed in the most advanced cases. The surface of the granules protruding through the perforations was surrounded by tapered epithelium in an attempt to protect the internal environment (Figure 4a,b) and eventually try to circumscribe the granules and eject them into the sinus cavity (Figure 4c,d).

The elevated space tends to regain its original position, whereas the graft material counteracts this tendency. It has been demonstrated both in animal [25,26,27,28] and human [29,30,31,32] studies that shrinkage depends on the material used. Once postsurgical edema is resolved, the SM collapses onto the periphery of the elevated space [33,34,35]. This generates a tight contact between the SM and xenograft granules that protrude beyond the boundary of the elevated space, establishing conditions for mucosal damage, such as mucosal thinning and perforations.

Thinned mucosae and perforations were observed after 2 weeks of healing, especially in the DBBM group, and were mostly located against the sharp edges of the biomaterial that protruded beyond the dome shape of the elevated space. At this stage, it might be argued that damage to the SM was triggered by the pressure elicited during the grafting procedures and by bleeding and edema within the elevated space, as described in experimental [33] and clinical studies [34,35,36,37,38,39,40]. Nevertheless, after four weeks of healing, fewer thinned and perforated mucosae were observed. Thus, it can be speculated that the pressure resulting from the grafting procedure was exhausted. However, the capability of the SM to circumscribe and eject the exposed granules should also be considered. Moreover, the slight decrease in the number of thinned mucosae observed in the 4-week period compared to the 2-week period might be ascribed to a perforation of the mucosa and the consequent expulsion of the involved granules and the subsequent healing of the mucosa between 2 and 4 weeks.

In the 2-week period, a higher number of thinned mucosae was present in DBBM No-Wn than in DBBM Wn. This might be related to the fact that the inward bone window occupied approximately one-third of the length of the elevated mucosa, so the mucosa was exposed less to contact with the granules than the control sites. However, four perforations in four different sinuses were observed at DBBM Wn, whereas only one was found in DBBM No-Wn. Moreover, the diameters of the perforations were larger in the former than in the latter. This, in turn, means that the trap-door technique may be more prone to triggering perforations during the first period of healing, which may be ascribed to technical procedures.

In the DBBM group during the 8-week period, a large increase in thinned mucosa sites was observed, which was higher in the No-Wn sites than in the Wn sites. Perforations were also higher in the former than in the latter. This might be interpreted as the bone lid providing a certain degree of protection.

**Figure 9:**
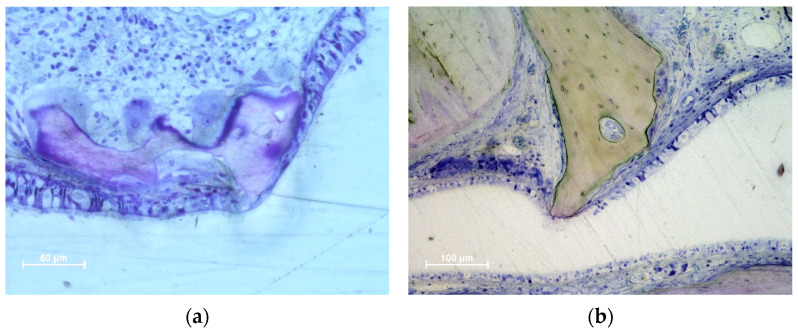
Photomicrographs of ground sections illustrating the different concentration of osteoclastic zone and the higher grade of resorption at the collagenated graft (**a**) compared to the DBBM graft (**b**). Toluidine blues stain.

In the collagenated group, the membrane adjacent to the SM occupied almost the entire length of the elevated mucosa during all healing periods, protecting against the damaging effect of the granules. In the collagenated groups, considering all periods of haling, a few thinned sites and only two perforations in two out of 36 sinuses were found. This could be ascribed to the higher resorption rate of the collagenated xenograft compared to DBBM, which decreased the number of contacts between the mucosa and the granules of the biomaterial, resulting in a low number of damaging events (Figure 9). The percentages reported in previously published articles of residual graft after 8 weeks of healing were 7.8–9.6% for the collagenated graft [22], and 28.3–42.4%, for the DBBM graft [23]. The higher content of osteoclastic zones and higher rate of resorption of the collagenated graft with respect to DBBM were also confirmed by another report [41]. The protective effect of the collagen membrane adjacent to SM could not be confirmed because of the low incidence of thinned mucosae and perforations in the collagenated group.

The increasing damaging effect of the granules on SM over time was also confirmed by the increased number of sites with a width of <10 µm in the DBBM. This finding also suggests the possible occurrence of further perforations over time.

Despite the ability of SM to heal the perforated sites, the presence of inflammatory infiltrates in these regions should be considered a risk to patient health. In fact, it has been reported that particles of biomaterial extruded inside the sinus triggered sinusitis [13,14,15,42,43], and in some cases, the biomaterial had to be removed [14,15]. Extrusion of the biomaterial might have occurred through a perforation of SM not recognized during the elevation, during the placement of the graft, or in later periods, as shown by the data reported in the present study. This agrees with the data reported in other studies [17,18,19]. In a rabbit model [17], progressive thinning and subsequent perforations of the SM in contact with sharp edges of slowly resorbable deproteinized bovine bone mineral (DBBM) were observed. In another similar experiment in rabbits [18], either a DBBM graft or autogenous bone was used as a filler, and simultaneous implant installation was carried out. A higher rate of thinned mucosae and perforations was observed in DBBM grafts than in autogenous bone grafts. This was related to the higher rate of resorption in the latter than that in the former. In another experiment [19], two slowly resorbable xenografts were compared. A similarly high rate of thinned mucosa sites and perforations was observed. The present study showed that the resorption rate affected the damage to the SM; the higher the resorption, the lower the damage. It should also be noted that the histological analyses were localized to the central region of the elevated space. It might be assumed that further thinned sites and perforations might have been present in the elevated regions outside the central region analyzed.

SM perforations with the extrusion of biomaterial granules have also been described during transcrestal sinus floor elevation (TSFE) in in vivo [11,12] and ex vivo human studies [44,45,46]. To reduce the risk of perforations, TSFE has been applied without the use of biomaterials [47,48,49]. Even though the implant success rate over time has been shown to be similar with or without grafts [6,50], higher vertical bone gain was reported for the former than for the latter [51,52].

The main limitation of the present study is the selection of two different experiments. However, these studies were selected to ensure homogeneity in methodology. They were performed by the same research team at the same research center. The healing periods were similar and histological slides were prepared by the same technician in the same laboratory using the same methods. All histological slides were analyzed by the same assessor. Other limitations include the smaller size of the maxillary sinus and thinner mucosa in rabbits than in humans. The pristine mucosa width in the present study ranged from 67–113 µm, while in humans, a width of 0.45–1 mm has been reported, as evaluated histologically [53]. Moreover, CBCT analysis revealed 30 of 88 sinuses with a width of <1 mm (min. 0.4 mm) [54]. It should not be excluded that over time, the thinnest SM might undergo thinning and eventually perforate when in contact with the sharpened edges of the non-reabsorbed graft.

The SM exhibited repair processes around the exposed graft to discharge it and re-establish the continuity of the mucosa. Granules inside the sinus might be cleared through the ostium and infundibulum. A pre-surgical CBCT [55] analysis of sinuses showed that 19 out of 72 sinuses presented an ostium diameter < 1.5 mm. It might be argued that granules exceeding this dimension might present difficulties in being easily cleared through the ostium/infundibulum and possibly induce obstruction.

## 5. Conclusions

More damage to the Schneiderian membrane was observed in the DBBM group than that in the collagenated group. The presence of the inward bone window offered protection from damage to the Schneiderian membrane.

## Data Availability

The data are available on reasonable request.
